# Wellens Syndrome Electrocardiogram Pattern in a Patient With Multi-Vessel Coronary Artery Disease

**DOI:** 10.7759/cureus.26418

**Published:** 2022-06-29

**Authors:** Aalok Parekh, Lam Nguyen, Lauren Wilke, Arminder Singh, Suresh Paudel, Yazan Khouri

**Affiliations:** 1 Internal Medicine, Cape Fear Valley Medical Center, Fayetteville, USA; 2 School of Medicine, Campbell University School of Osteopathic Medicine, Lillington, USA; 3 Internal Medicine, Campbell University School of Osteopathic Medicine, Lillington, USA; 4 Cardiology, Cape Fear Valley Medical Center, Fayetteville, USA

**Keywords:** cardiac catheterization, coronary artery bypass graft, non-st-elevation myocardial infarction, left anterior descending, wellens syndrome

## Abstract

Wellens syndrome is an electrocardiogram (ECG) pattern associated with critical stenosis of the proximal left anterior descending (LAD) artery. In patients with Wellens syndrome, characteristic biphasic or inverted T waves are seen on ECG. This case report presents a 48-year-old male admitted for chest pain and shortness of breath who was found to have a Wellens syndrome ECG pattern. Oddly, cardiac catheterization revealed multi-vessel coronary artery disease, and critical proximal LAD artery stenosis was not appreciated. Ultimately, the patient was treated with coronary artery bypass graft (CABG) surgery and later discharged in stable condition.

## Introduction

Wellens syndrome is an electrocardiogram (ECG) pattern that was first characterized in 1982 by Wellens’ group, describing acute ECG changes associated with critical stenosis of the proximal left anterior descending (LAD) artery [1]. Patients with Wellens syndrome are found to have biphasic or deeply inverted T waves in leads V2-V3 [1-5]. Laboratory findings typically reveal normal to slightly elevated cardiac biomarkers. Wellens syndrome patients can present asymptomatically or with symptoms similar to acute coronary syndrome, including exertional chest pain or tightness, relieved with rest. This report describes a patient presenting with a Wellens syndrome ECG pattern; however, he was later diagnosed and treated for multi-vessel coronary artery disease.

## Case presentation

A 48-year-old male with a past medical history of post-traumatic stress disorder, attention-deficit hyperactivity disorder, unspecified bipolar disorder, polysubstance abuse, extensive tobacco use, and hypertension returned to the hospital for non-ST-elevation myocardial infarction (NSTEMI) evaluation after he had left against medical advice earlier that day, citing improvement in his presenting symptoms. One day prior to his readmission, the patient had presented to the emergency department with complaints of dyspnea and left-sided chest pain radiating to the left arm for one hour. The patient was contacted and advised to come back due to elevated troponin, and he was readmitted for further work-up of possible NSTEMI upon his return. At his initial presentation, he was found to have elevated troponin I with a maximum value of 13.600 ng/ml, and his urine drug screen was positive for cocaine, marijuana, and opiates. His initial electrocardiogram showed sinus rhythm with an old inferolateral infarct and no acute ST changes.

**Figure 1:**
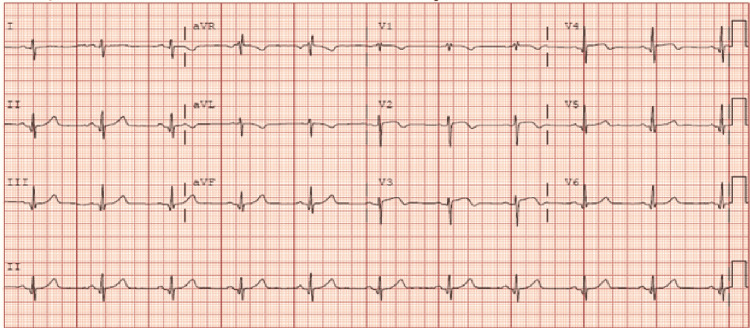
ECG demonstrating biphasic T waves in leads V2-V3 and old anterolateral infarct as well as minimal ST elevation in anterior leads.

**Figure 2:**
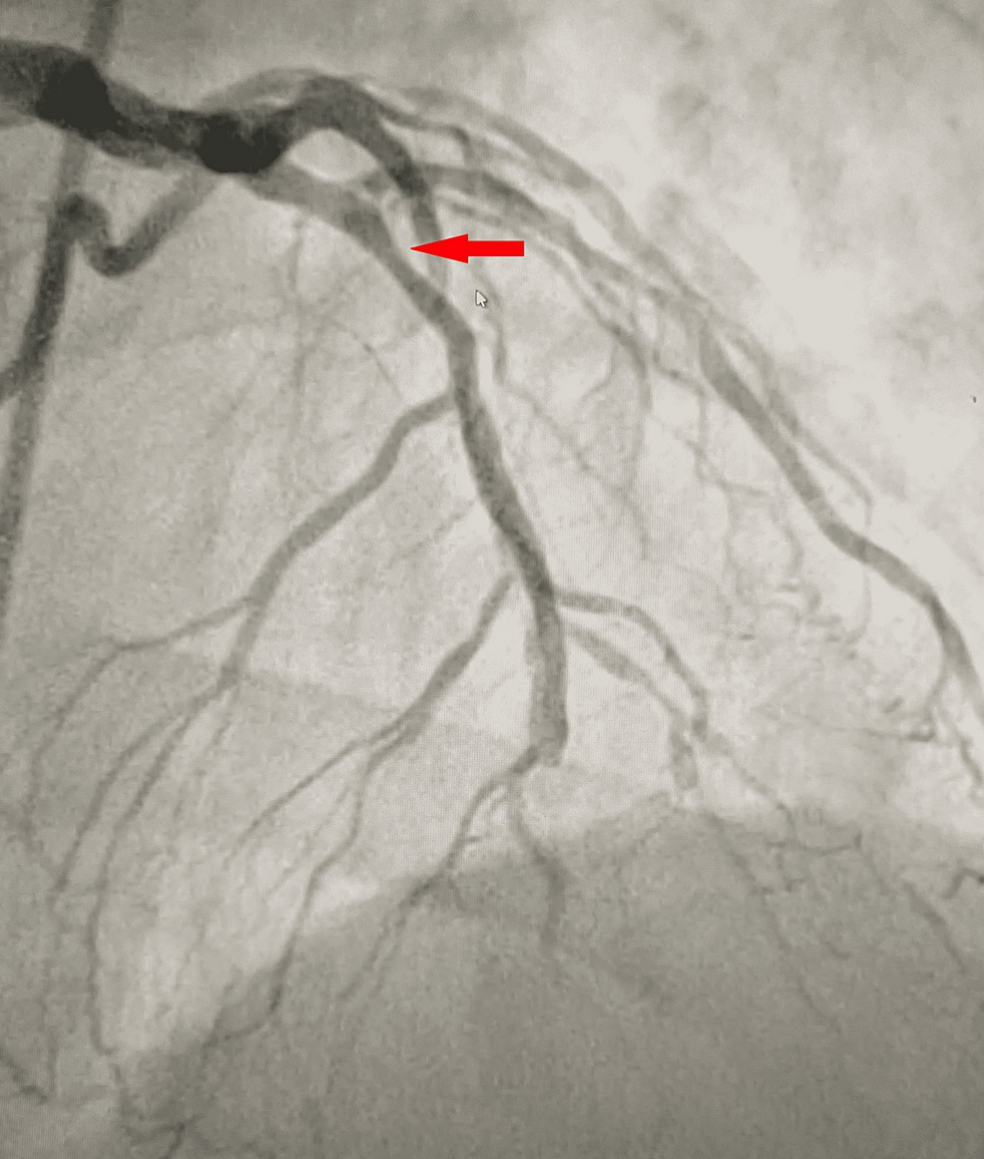
Coronary angiogram demonstrating 60% stenosis of the mid-left anterior descending artery. Obstruction is demonstrated by the red arrow.

**Figure 3:**
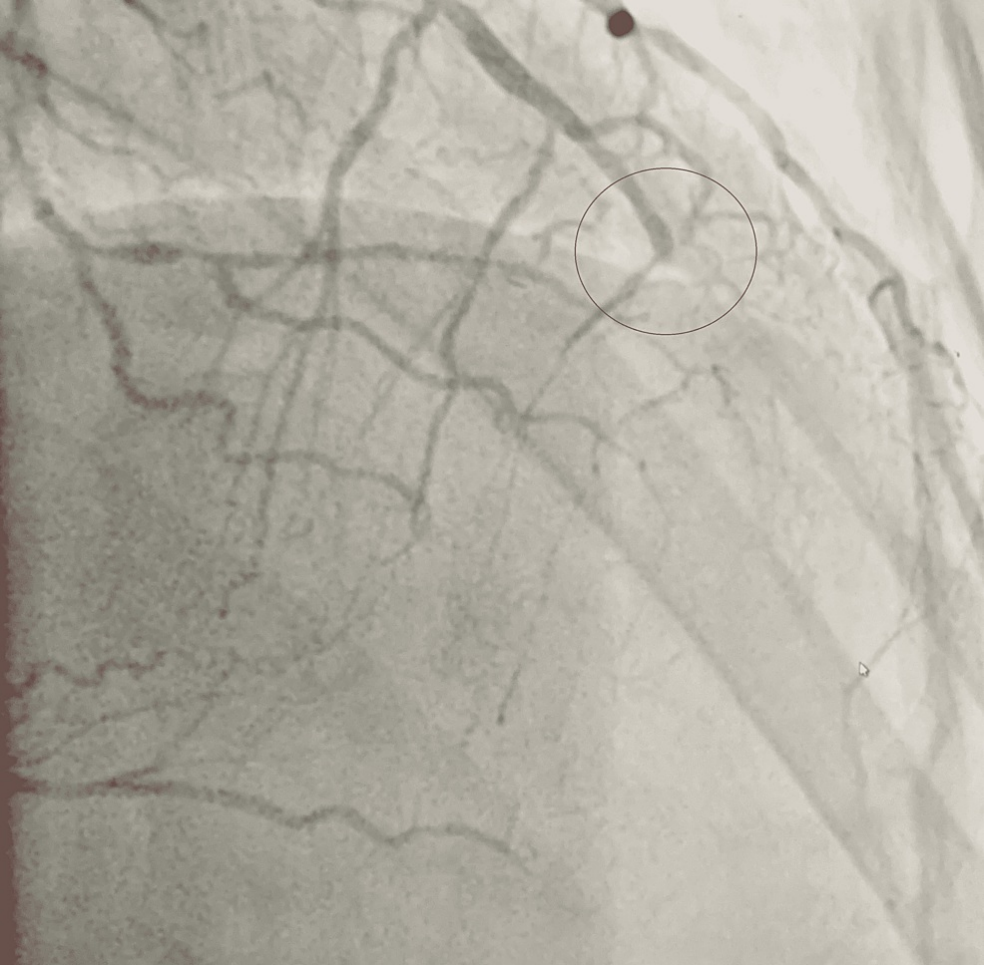
Coronary angiogram demonstrating total occlusion of the distal left anterior descending artery. Obstruction is demonstrated by the red circle.

**Figure 4:**
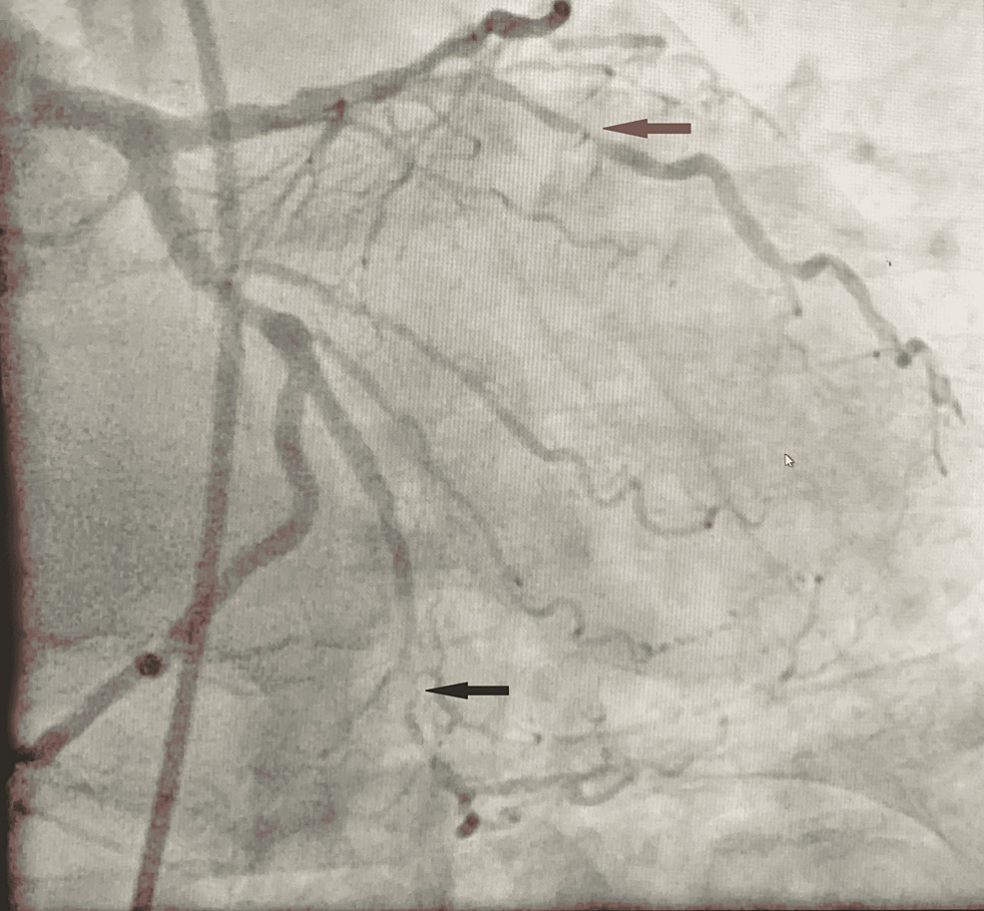
Coronary angiogram demonstrating 90% stenosis of the first obtuse marginal branch (demonstrated by the black arrow) and 70% stenosis of the first diagonal branch (demonstrated by the red arrow).

**Figure 5:**
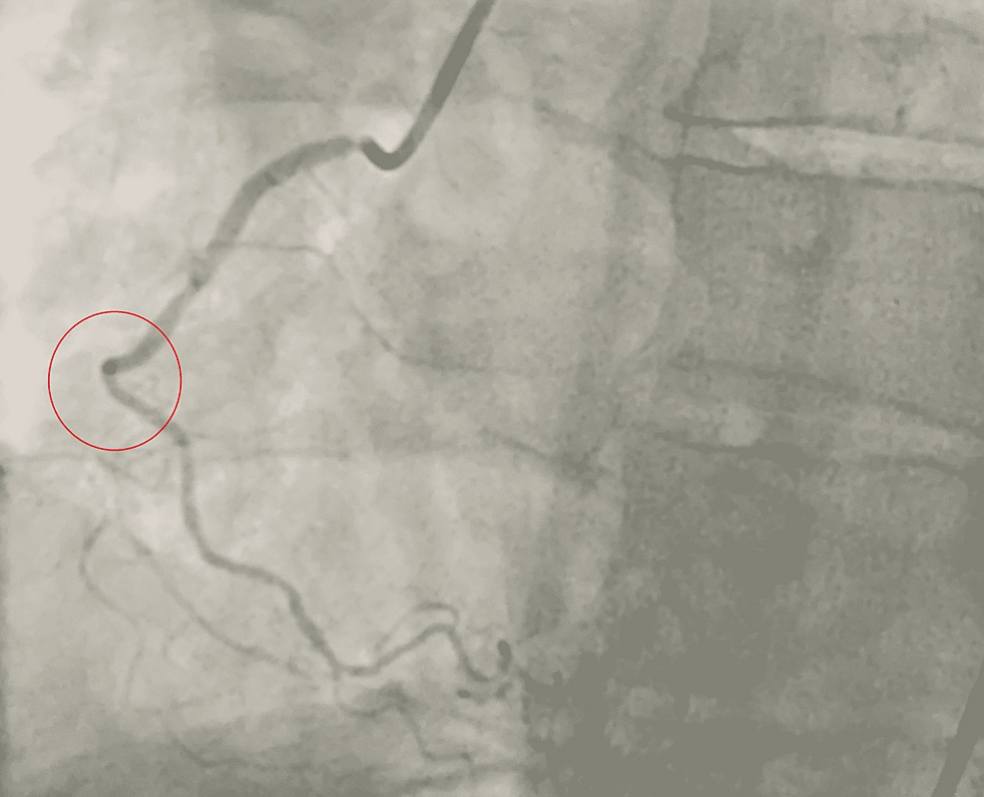
Coronary angiogram demonstrating total occlusion of the right coronary artery. Obstruction is demonstrated by the red circle.

**Figure 6:**
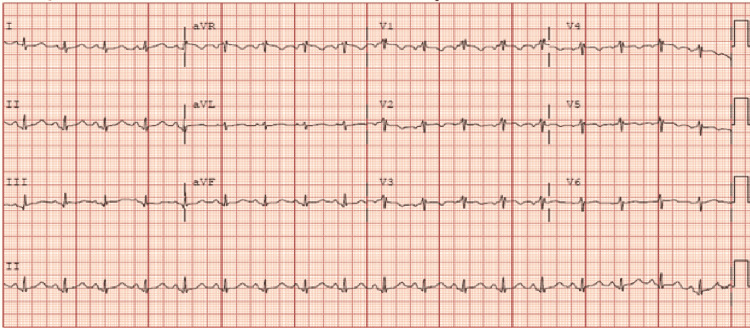
Post-coronary artery bypass graft surgery ECG demonstrating resolution of the biphasic T waves.

Upon the patient’s readmission, his troponin I remained elevated, and a repeat ECG also showed sinus rhythm with biphasic T waves in leads V2-V4 in addition to the above-mentioned (Figure 1). Cardiac catheterization was performed on day 2, demonstrating significant multi-vessel coronary artery disease including 60% stenosis of the mid-left anterior descending artery, 100% of the distal left anterior descending artery, 60% of the left circumflex artery, 90% of the first obtuse marginal artery, 70% of the diagonal branch, and 100% of the right coronary artery with collateral flow from left. A transthoracic echocardiogram was performed, showing a left ventricular ejection fraction of 40-45%, and an intra-aortic balloon pump was also placed due to ongoing chest pain during the catheterization (Figures 2-5). On day 4 of his admission, a coronary artery bypass graft (CABG) surgery was performed for his coronary artery disease. The patient's hospital course was complicated by prolonged bleeding following the CABG surgery, which was resolved with an additional exploratory surgery on day 5. Fortunately, the patient made a gradual improvement and was ultimately discharged in stable condition three weeks after his surgery (Figure 6).

## Discussion

Risk factors for Wellens syndrome are similar to those for CAD including hypertension, diabetes, dyslipidemia, obesity, sedentary lifestyle, and smoking [4,5]. Wellens syndrome is triggered by a transient obstruction of the LAD coronary artery. This results from atherosclerotic plaque rupture causing LAD occlusion, which is later resolved due to clot lysis or other occlusion disruption before myocardial infarction has happened. Therefore, Wellens syndrome is thought of as a pre-infarction state. It is not completely understood why Wellens syndromes produce their characteristic ECG changes, but coronary artery spasms and stunned cardiomyocytes are thought to be the reasons. Transmural ischemia-reperfusion is also thought to cause myocardial edema, which leads to ECG changes.

There are two T wave patterns seen in patients with Wellens syndrome, type-A and type-B [4]. Type-A T waves are seen in 25% of cases, and they are biphasic waves that start with a positive deflection and end with a negative deflection in leads V2 and V3. Deeply inverted and symmetrical T waves are found in the type-B pattern, and they account for 75% of the cases. These inverted T waves are typically seen in V2 and V3, but they can also be present in leads V1, V4, V5, and V6. As the disease progresses, type-A T waves can evolve into type-B T waves. As a pre-infarction state, Wellens syndrome can develop over a span of days to weeks. ECG patterns can start to develop when patients are pain-free. After patients experience chest pain, acute ST-segment and T wave changes can also be seen. The diagnosis of Wellens syndrome can be made with either type-A or type-B T waves in addition to isoelectric or minimally elevated ST segments of less than 1 mm, mildly elevated or normal cardiac biomarkers, and preservation of R wave progression in precordial leads.

When Wellens syndrome is suspected or diagnosed, interventional cardiology consultation is recommended. In the meantime, patients can be temporarily managed with medical treatment of nitrates, heparin, aspirin, statin, and beta-blockers. Because the LAD is critically stenosed, large myocardial infarctions are commonly seen in these patients within weeks of presentation. For that reason, patients tend to do poorly with medical therapy alone. It is crucial to note that stress tests are contraindicated in these patients for the same reason. As a result, interventional revascularization treatments are needed to definitively treat Wellens syndrome patients. Percutaneous coronary intervention (PCI) is usually the treatment of choice instead of CABG, as data has shown PCI has similar survival benefits as CABG when used in patients with LAD artery disease [4,6].

Our patient presented with typical signs and symptoms of Wellens syndrome, including chest pain, chest tightness, shortness of breath, and biphasic T waves in leads V2 and V3. However, the troponin levels were markedly elevated, and the cardiac catheterization showed multi-vessel disease with moderately stenosed mid-LAD. This disqualified the patient from the diagnosis of Wellens syndrome as defined above. Due to the concomitant stenosis of the left circumflex and right coronary arteries, a reduction of collateral blood flow to the anterior wall was thought to be the reason that predisposed anterior cardiomyocytes to ischemia. This helps to explain the appearance of biphasic T waves in the patient who had a lesser degree of LAD stenosis. With this, would it be unreasonable to think of the patient's ECG presentation as an induced Wellens syndrome with moderately stenosed LAD precipitated by multi-vessel stenosis?

## Conclusions

Accurate recognition and diagnosis of Wellens syndrome are crucial in presenting patients, as it can quickly turn into myocardial infarction. It is also important to initiate medical treatments to stabilize patients, in addition to consultations for cardiology evaluation and intervention. Moreover, in patients with a Wellens syndrome ECG pattern and elevated cardiac biomarkers as demonstrated in this case, a significant multi-vessel coronary disease with some degree of LAD stenosis should be considered and investigated in a timely manner. This case study also documents the findings and effective management of a patient with multi-vessel coronary disease presenting with a Wellens syndrome ECG pattern.
